# Stability-driven multi-omics integration for reproducible latent structure

**DOI:** 10.64898/2026.06.23.734064

**Published:** 2026-06-27

**Authors:** Haibin Guan, Maaike van Gerwen, Seunghee Kim-Schultz, Elena Colicino, Georgia Dolios, Lauren M. Petrick

## Abstract

High-dimensional multi-omics data integration offers novel opportunities to characterize complex biological systems. Even though sampling variability frequently compromises findings, particularly in small cohorts, the reproducibility and generalizability of the derived latent structures are insufficiently evaluated. We propose a Stability-driven framework for multi-omics integration that combines sparse generalized canonical correlation analysis with repeated cross-validation, out-of-sample projection, and systematic evaluation of both component-level and feature-level stability. We apply this framework to untargeted metabolomic and Olink targeted inflammation proteomic profiles in a thyroid cancer case-control cohort (n = 162). Our Stability-driven integration identified reproducible metabolomic and proteomic latent components that showed consistent out-of-sample disease associations and tracked temporally structured changes relative to time to diagnosis. The proposed framework provides a generalizable strategy for identifying reproducible latent structures that improve robustness of biological inference in multi-omics studies.

## Full Text Availability

The license terms selected by the author(s) for this preprint version do not permit archiving in PMC. The full text is available from the preprint server.

